# Silencing of CXCR4 sensitizes triple-negative breast cancer cells to cisplatin

**DOI:** 10.18632/oncotarget.2741

**Published:** 2014-12-11

**Authors:** Sixian Liang, Xun Peng, Xiaoli Li, Ping Yang, Linhao Xie, Yaochen Li, Caiwen Du, Guojun Zhang

**Affiliations:** ^1^ Department of Breast Medical Oncology, Cancer Hospital of Shantou University Medical College, Shantou 515031, PR China; ^2^ Department of Radiotherapy, Cancer Hospital of Shantou University Medical College, Shantou 515031, PR China; ^3^ The Breast Center, Cancer Hospital of Shantou University Medical College, Shantou 515031, PR China

**Keywords:** CXCR4, triple-negative breast cancer, cisplatin, chemosensitivity, apoptosis

## Abstract

Triple-negative breast cancer (TNBC) is an aggressive form of breast cancer for which there is no effective treatment. Previously, we and others demonstrated that CXCR4 surface expression is an independent prognostic factor for disease relapse and survival in breast cancer. In this study, we investigated the effects of CXCR4 gene silencing on cisplatin chemosensitivity in human triple-negative breast cancer cell lines. We found that CXCR4 silencing significantly inhibited cell growth, decreased colony formation, and enhanced cisplatin sensitivity while overexpression of CXCR4 rendered cells more resistant to cisplatin. Moreover, the percentage of apoptosis and cell cycle arrest at the G2/M phase of cisplatin-treated CXCR4 knockdown cells was significantly higher than control cells. Furthermore, we demonstrated CXCR4 knockdown cells showed lower levels of mutant p53 and Bcl-2 protein than the control group, while also having higher levels of caspase-3 and Bax. However overexpression of CXCR4 had the reverse effect. *In vivo* experiments confirmed that downregulation of CXCR4 enhanced cisplatin anticancer activity in tumor-bearing mice, and that this enhanced anticancer activity is attributable to tumor cell apoptosis. Thus, this study indicates that CXCR4 can modulate cisplatin sensitivity in TNBC cells and suggests that CXCR4 may be a therapeutic target for TNBC.

## INTRODUCTION

Triple-negative breast cancer (TNBC) is an invasive carcinoma, of the breast, that lacks expression of the estrogen receptor (ER), progesterone receptor (PR) and human epidermal growth factor receptor 2 (HER2). TNBC accounts for about 12–17% of all breast cancers and is aggressive: there is no targeted therapy available. Patients with TNBC have an increased likelihood of distant recurrence (brain, lung and liver) and of death compared to women with other types of breast cancer [1, 2]. In addition to surgery, systematic chemotherapy like cisplatin plays an important role in TNBC treatment, especially for patients with advanced TNBC disease [3, 4].

Patients usually have a good initial response to cisplatin-based chemotherapy. However, drug resistance is a fundamental problem in TNBC management, and is responsible for most cases of treatment failure in patients with metastatic cancer [5, 6]. Ciplatin's cytotoxicity to normal tissues and cancer cells's acquired resistance reduces the clinical efficacy of this drug [7]. The exact mechanism of cisplatin resistance is not clear. Previous studies have shown that the development of drug resistance is related to the tumor microenvironment, in which chemokines appear to play pivotal roles in tumor progression, metastasis and tumor cell dormancy [8, 9].

To date, 46 different human chemokines are described as ligands for at least 18 G protein–coupled receptors [10]. In the treatment of acute myeloid leukemia (AML), interrupting the connection between leukemic cells and the tumor microenvironment by targeting the stromal-derived factor-1α/CXCR4 (SDF-1α/CXCR4) axis has become an attractive approach [11, 12]. Previously, we and others demonstrated that CXCR4 surface expression is an independent prognostic factor for disease relapse and survival in breast cancer [13]. CXCR4 is of particular importance in other solid cancers, including gastric cancer and colorectal cancer [14]. In addition, CXCR4 is now known to be critical in almost all aspects of cancer biology, including proliferation, apoptosis, invasion, metastasis and angiogenesis. In recent years, studies revealed that CXCR4 signaling mediates chemoresistance in hematologic malignancies [15].

Based on this information, we investigated the involvement of CXCR4 in TNBC sensitivity to cisplatin. To confirm whether CXCR4 affects the efficacy of treatment with cisplatin, we selected a stable CXCR4 knockdown TNBC cell line MDA-MB-231 and overexpression CXCR4 in MDA-MB-468 cells. *In vivo* and *in vitro* analyses demonstrated that CXCR4 knockdown cells enhanced the sensitivity to cisplatin, and that suppressing CXCR4 signaling may render TNBC cells responsiveness to cisplatin treatment. In contrast, overexpression of CXCR4 stimulated TNBC cell growth and enhanced resistance to cisplatin compared with that of control cells. We further examined whether CXCR4 knockdown increased cisplatin-induced apoptosis. The mechanism by which CXCR4 silencing induced apoptosis was also explored. In the present study, we aimed to reveal the potential role of CXCR4 in response to cisplatin and provide a new clue for future clinical treatments of TNBC patients who are resistant to cisplatin treatment.

## RESULTS

### CXCR4 knockdown enhances cisplatin-induced growth and colony formation inhibition in TNBC cells

Our previous studies indicated that CXCR4 silencing affects the proliferation of breast cancer cells compared with parental cells [13]. To assess the effects of CXCR4 on chemosensitivity, cell viability was assessed 48 h after exposure to various concentrations of cisplatin. We established stable cell lines that MDA-MB-231 shRNA-mediated knockdown of CXCR4 and transient transfection overexpression CXCR4 in MDA-MB-468 cells.

**Figure 1:**
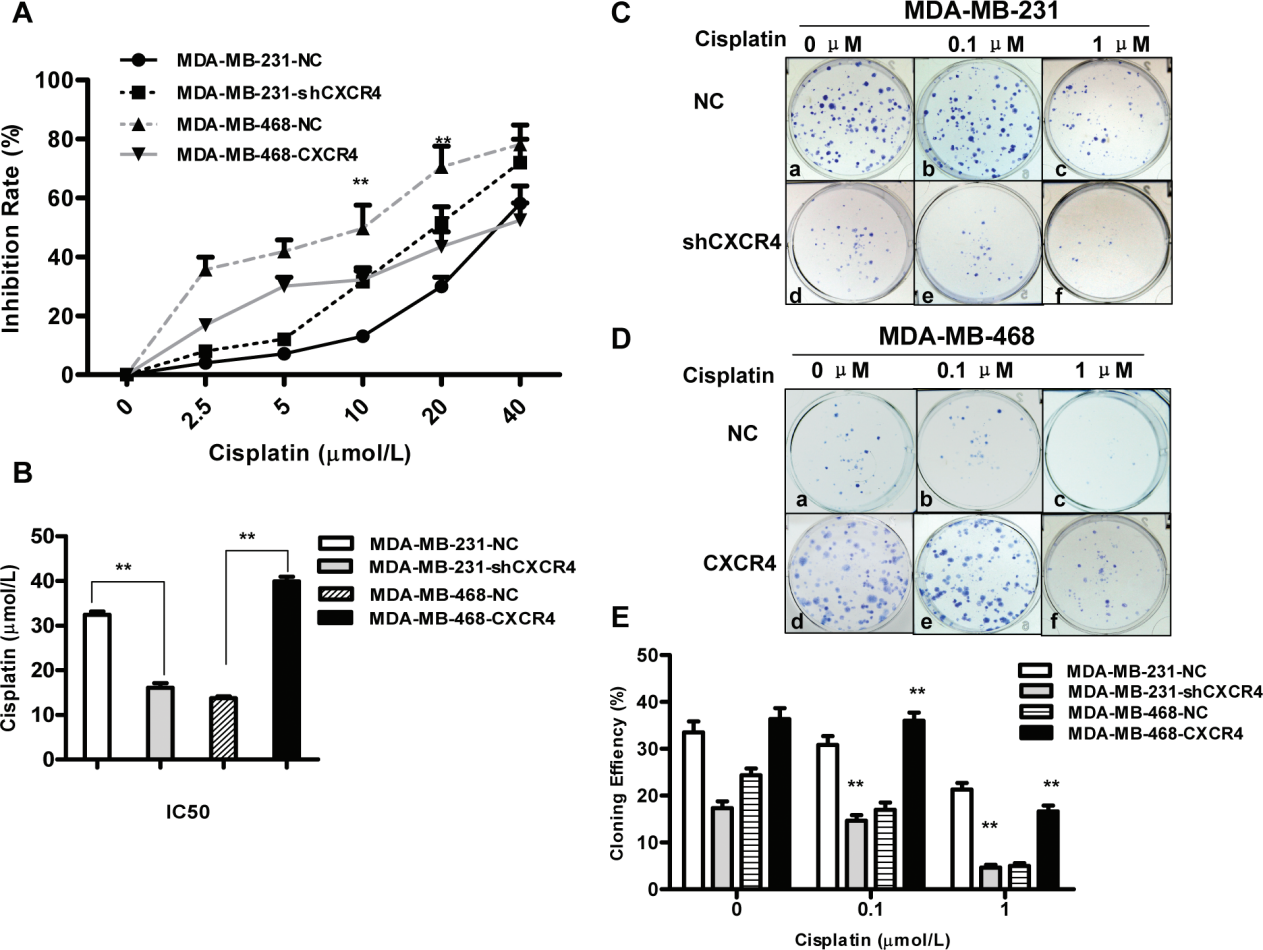
Effects of cisplatin on proliferation and colony formation in triple-negative breast cancer (TNBC) cells **(A)** CXCR4 knockdown and CXCR4 overexpression cells were treated with cisplatin for 48 h. The effect of CXCR4 on cisplatin sensitivity was measured by MTT assay. **(B)** IC_50_ values for cisplatin in MDA-MB-231 and MDA-MB-468 cells for 48 h were calculated by regression analysis using SPSS software based on the results of the MTT assays. **(C)** Colony formation of MDA-MB-231-NC and MDA-MB-231-shCXCR4 cells following treatment with cisplatin (0, 0.1 and 1 μM) for 48 h. **(D)** Colony formation of MDA-MB-468-NC and MDA-MB-468-CXCR4 cells following treatment with cisplatin (0, 0.1 and 1 μM) for 48 h. **(E)** The percentage of colony formation of MDA-MB-231 and MDA-MB-468 cells treatment with cisplatin. Data are represented as the mean ± *SD* of triplicate determinations. Each assay was performed in triplicate and repeated at least three times. *p < 0.05, **p < 0.01, as compared with untreated cells.

Suppression of CXCR4 increased sensitivity to cisplatin-mediated growth inhibition compared with the same cells transfected with empty vector (Figure 1A). Increased growth inhibition compared with MDA-MB-231-NC cells was observed at cisplatin concentrations of 10 and 20 μM. The half-maximal inhibitory concentration (IC_50_) (Figure 1B) at 48 h was 16.07 ± 1.77 μM and 32.43 ± 1.21 μM for MDA-MB-231-shCXCR4 and MDA-MB-231-NC cells, respectively (*p* = 0.000), indicating that CXCR4 knockdown increases sensitivity to cisplatin. Consistent with this, we found that it was more resistant to cisplatin in CXCR4 overexpression MDA-MB-468-CXCR4 cells than MDA-MB-468-NC cells. The IC_50_ at 48 h was 39.92 ± 1.8 μM and 13.73 ± 0.75 μM for MDA-MB-468-CXCR4 and MDA-MB-468-NC cells, respectively (*p* = 0.000, Figure 1B). In a colony formation assay, knockdown of CXCR4 inhibited MDA-MB-231 colony formation, whereas overexpression of CXCR4 stimulated MDA-MB-468 colony formation compared to the control (Figure 1C–1E). In addition, cisplatin effectively inhibited colony formation of these cells in a dose-dependent manner (*p* = 0.000). These results suggest that CXCR4 might be involved in the regulation of cell proliferation.

### CXCR4 knockdown increases cisplatin-induced apoptosis and G2/M phase arrest

**Figure 2:**
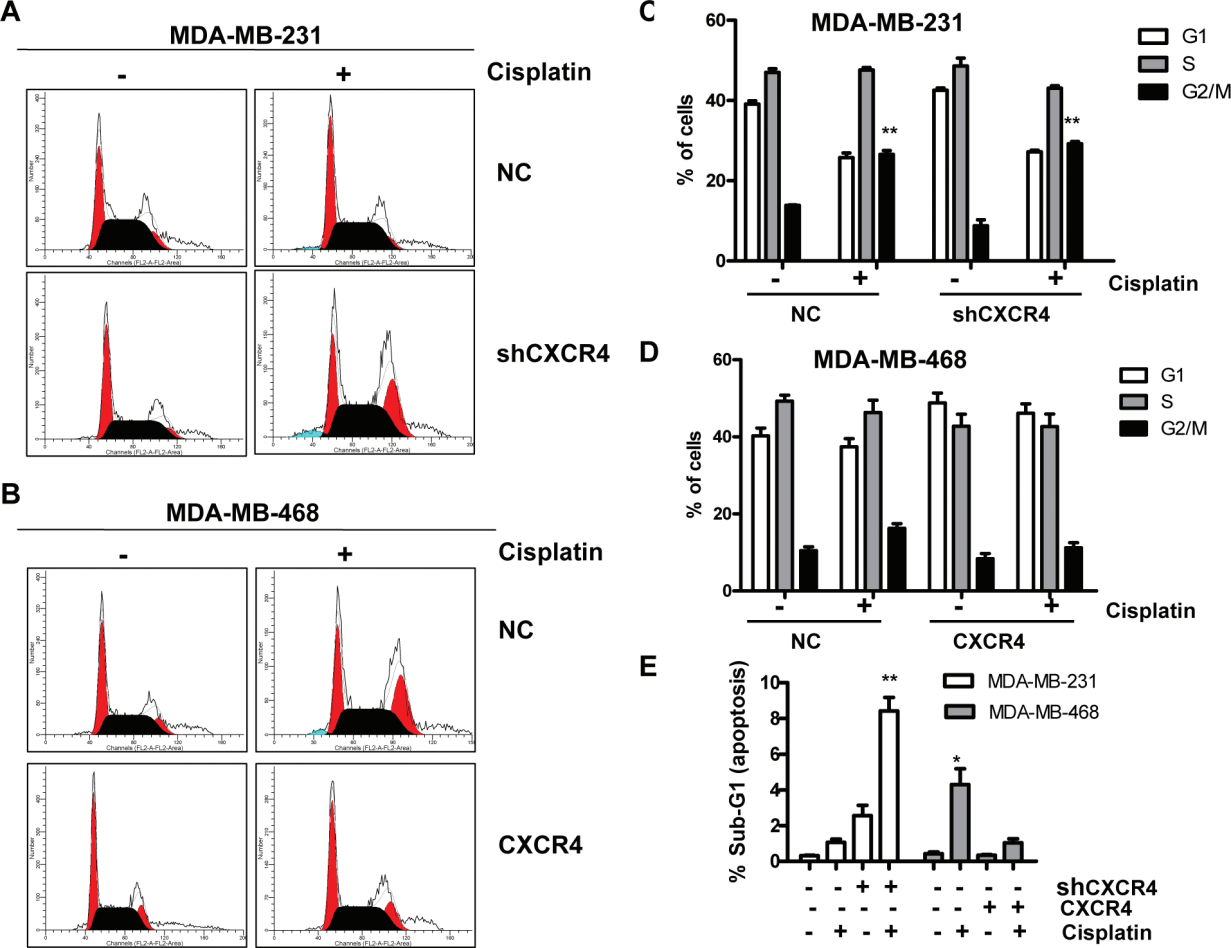
Flow cytometric analysis of the cell cycle phase distribution **(A)** DNA content of MDA-MB-231-NC and MDA-MB-231-shCXCR4 cells treated with cisplatin (0, 1 μM) was analyzed by propidium iodide (PI) staining. **(B)** DNA content of MDA-MB-468-NC and MDA-MB-468-CXCR4 cells treated with cisplatin (0, 1 μM) was analyzed by propidium iodide (PI) staining. **(C)** The effect of CXCR4 knockdown on cell cycle in MDA-MB-231 cells. From the left to right, the phases of the cell cycle were G1-phase, S-phase, and G2/M-phase. **(D)** The effect of CXCR4 overexpression on cell cycle in MDA-MB-468 cells. From the left to right, the phases of the cell cycle were G1-phase, S-phase, and G2/M-phase. **(E)** After treatment with 0 and 1 μM cisplatin for 48 h, the fraction of apoptotic cells was analyzed by flow cytometry. *p < 0.05, **p < 0.01, as compared with untreated cells.

To further assess the effect of CXCR4 on the sensitivity of TNBC cells to chemotherapy, we examined the cell cycle using flow cytometry (Figure 2A and 2B). The percentage of cells in each cell phase is shown in Figure 2C and 2D. Cisplatin (1 μM) treatment for 48 h increased the number of MDA-MB-231-shCXCR4 cells in G2/M phase compared with the untreated group (*p* = 0.001). To quantify apoptosis, we analyzed the percentage of sub-G1 cell cycles. The percentage of apoptosis of MDA-MB-231-shCXCR4 cells was 8.42 ± 0.76%, following treatment with 1 μM cisplatin. However, the same cisplatin concentration induced apoptosis in only 1.06 ± 0.18% of MDA-MB-231-NC cells (*p* = 0.002; Figure 2E). The percentage of cisplatin-induced apoptosis of CXCR4-negative MDA-MB-468 cells was higher than that of the control groups (4.30 ± 0.89% and 1.04 ± 0.22%, respectively, *p* = 0.025). However, there was no significantly observation of the G2/M arrest in MDA-MB-468-CXCR4 cells after cisplatin treatment compared with the untreated group (*p* = 0.162).

### CXCR4 knockdown increases the sensitivity of TNBC cells to cisplatin *in vivo*

**Figure 3:**
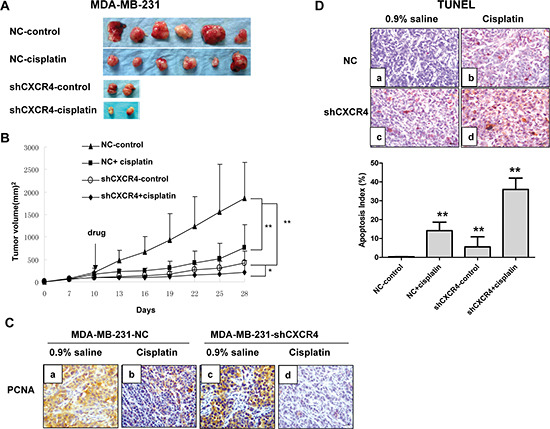
CXCR4 knockdown sensitizes TNBC to cisplatin *in vivo* **(A)** Gross appearance. Gross appearance and the primary tumors of each group are shown. **(B)** Growth curves of tumors for MDA-MB-231-NC and MDA-MB-231-shCXCR4 with cisplatin or 0.9% saline. **(C)** Immunohistochemical staining of xenograft tumors for PCNA (magnification = 40×). **(D)** Representative pictures of TUNEL-positive staining (brown) for MDA-MB-231-NC and MDA-MB-231-shCXCR4 xenografts. Apoptosis index was calculated as the number of apoptotic cells divided by the total number of tumor cells. *p < 0.05, **p < 0.01, as compared with untreated cells.

We further analyzed whether a similar phenomenon could be observed *in vivo* using an immunodeficient nude mouse model (Figure 3A). We found that tumors derived from MDA-MB-231-shCXCR4 cells showed slower growth and smaller tumor volume and weight 28 days after implantation, compared to tumors derived from MDA-MB-231 cells. MDA-MB-231-shCXCR4-injected mice that were treated with cisplatin displayed a 6-fold decrease in tumor weight after 4 weeks, compared with the untreated group (*p* = 0.02; Figure 3B). Furthermore, subcutaneous tumors from MDA-MB-231-injected mice showed increased cell proliferation as indicated by the strong positive staining of PCNA, a marker for cell proliferation, compared with MDA-MB-231-shCXCR4 mice and mice treated with cisplatin (Figure 3C a–d).

### CXCR4 knockdown increases cisplatin-induced apoptosis *in vivo*

TUNEL staining of tumors showed a brown positive signal located in the nucleus and concentrated in the nucleoplasm close to the nuclear membrane. We observed TUNEL-positive cells with different staining levels in each group. The AI percentage was 36.00 ± 13.0% in the MDA-MB-231-shCXCR4 cisplatin-treated group and 14.16 ± 4.50% in the saline-treated group. The AI percentage for the MDA-MB-231 cells treated with cisplatin and saline was 5.58 ± 5.30% and 0.3 ± 0.1%, respectively. Therefore the AI was significantly higher in the CXCR4 sh-RNA and cisplatin combination group than in the other three groups (*p* = 0.026; Figure 3D).

### CXCR4 knockdown enhances cisplatin-induced apoptosis through the Bax/Bcl-2/caspase-3 pathway

**Figure 4:**
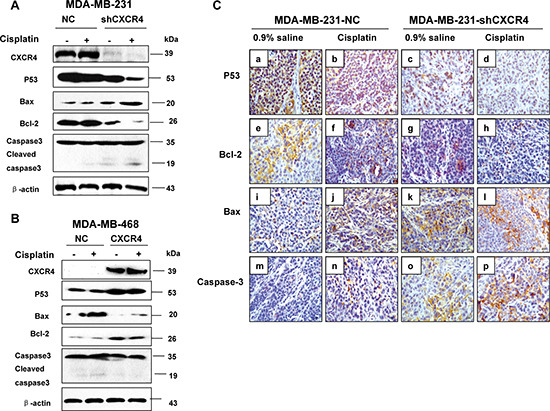
The effect of CXCR4 on protein expression and cell cycle associated proteins in TNBC cells **(A)** MDA-MB-231-NC and MDA-MB-231-shCXCR4 cells were treated with cisplatin (0, 10 μM) for 48 h, cellular protein was harvested, and Western blot analysis was performed to investigate the levels of mutant p53, Bax, Bcl-2 and caspase-3. The β-Actin was detected as a loading control. **(B)** MDA-MB-468-NC and MDA-MB-468-CXCR4 cells were treated with cisplatin (0, 10 μM) for 48 h, cellular protein was harvested, and Western blot analysis was performed to investigate the levels of mutant p53, Bax, Bcl-2 and caspase-3. The β-Actin was detected as a loading control. **(C)** Immunohistochemical staining of xenograft tumors for p53, Bax, Bcl-2 and caspase-3 (magnification = 40×).

To further understand the molecular events involved in the apoptosis resulting from CXCR4 knockdown, we next investigated the expression of p53, Bcl-2, Bax, and caspase-3, which are pivotal for cell apoptosis. Figure 4A shows the expression of these proteins in MDA-MB-231 cells. Expression of mutant p53 was clearly downregulated in MDA-MB-231 cells after transfection of CXCR4 shRNA and/or treatment with cisplatin. Simultaneously, we observed upregulation of Bax and cleaved caspase-3 and downregulation of Bcl-2, suggesting that a decrease in the Bcl-2/Bax ratio might be involved in the apoptosis induced by CXCR4 shRNA combined with cisplatin in MDA-MB-231 cells. Furthermore, the expression of mutant p53 and Bcl-2 were increased and decreased Bax was decreased in CXCR4 transfected MDA-MB-468 cells (Figure 4B). But no observation of cleaved caspase-3 in MDA-MB-468-CXCR4 cells after treatment with cisplatin.

We then used immunohistochemistry and detected p53, caspase-3, Bcl-2, and Bax expression in tumor tissues obtained from the orthotopic mouse model. We similarly found results in this model, with significantly decreased p53 and Bcl-2 proteins and increased caspase-3 and Bax proteins (Figure 4C a–p). These findings demonstrated that CXCR4 down-regulation increased the apoptosis via leading to the imbalance in the expression of apoptosis related proteins.

## DISCUSSION

Triple-negative breast cancer (TNBC) is a devastating disease for which there is no effective treatment. In addition to surgery, chemotherapy is a crucial element of treatment for TNBC patients. Cisplatin is widely accepted as a palliative treatment [16]. Despite improvements in the treatment of advanced TNBC, cisplatin resistance is a fundamental problem in TNBC management, responsible for most cases of treatment failure in patients with metastatic TNBC [17–20]. An emerging understanding of the molecular pathways that characterize cell growth, cell cycle, apoptosis, angiogenesis and invasion have provided novel targets for TNBC therapy [21, 22]. Combining cisplatin with non-cytotoxic agents is imperative to improving the efficacy of chemotherapeutics and overcoming cisplatin-resistance [23]. Previous studies reported that treating leukemia with a combination of CXCR4 inhibitors and chemotherapeutic agents produced additive therapeutic effects [24, 25]. However, the exact mechanism underlying cisplatin-resistance is still unclear.

CXCR4 is a seven transmembrane, G protein-coupled receptor widely expressed in various cell types, including lymphocytes, hematopoietic stem cells, endothelial and epithelial cells, and cancer cells [26]. Several studies have demonstrated the involvement of CXCR4 in cell proliferation, migration, and metastasis of solid tumors in a variety of cancers, such as gastric cancer, breast cancer and colorectal cancer [27–29]. CXCR4-positive cell is an independent prognostic factor for poor patient survival: CXCR4 expression is associated with tumorigenesis and progression [30, 31]. Our previous studies indicated that CXCR4 was expressed more frequently in the TNBC than in other subtypes breast cancer. In the TNBC group, CXCR4 positive patients have a significantly higher rate of visceral metastasis (liver, lung and brain). The expression level of CXCR4 also significantly correlates with tumor size, advanced TNM stage, and shorter overall and disease-free survival [13].

It was reported that patients with higher CXCR4 expression have significantly lower chemosensitivity and poorer progression-free and overall survival. Knocking down CXCR4 via small interfering RNA (siRNA) suppresses cell proliferation, and increases apoptosis and chemosensitivity to cisplatin in epithelial ovarian cancer [32]. Mao et al. reported that CXCL12-combining CXCR4 receptors could inhibit caspase-3 activation by increasing the ratio of Bcl-2/Bax after traumatic brain injury: this inhibition protected neurons from apoptosis [33]. Another study demonstrated that ADM3100, a CXCR4 antagonist, could inhibit cell migration, decrease the secretion of angiogenic cytokines, and downregulate the Bcl-2/Bax ratio to modulate apoptosis progression in bone marrow mesenchymal stem cells (MSC) [34].

In the current work, we show that downregulation of CXCR4 results in increased cisplatin-induced growth inhibition. Consistent with this, we found that it was more resistant to cisplatin in CXCR4 overexpression MDA-MB-468-CXCR4 cells than MDA-MB-468-NC cells. Interestingly, we find that knockdown of CXCR4 not only has an inhibitory effect on TNBC, but can also dramatically enhance the chemosensitivity to cisplatin. Analysis of the percent of cells in the sub-G1 cell cycle phase, to quantify apoptosis, revealed that the percentage of cells in apoptosis and G2/M cell cycle arrest for MDA-MB-231-shCXCR4 cells treated with cisplatin was significantly higher than for control cells.

To explore the mechanisms underlying this phenomenon, we examined the expression levels of p53, Bax, Bcl-2 and caspase-3. In this study, the expression of mutant p53 was downregulated in MDA-MB-231-shCXCR4 cells at the protein level after cisplatin treatment. Simultaneously, we observed upregulation of Bax and downregulation of Bcl-2 while overexpression of CXCR4 had the reverse effect. These findings suggest that decreased Bcl-2/Bax ratios might be involved in the apoptosis induced by CXCR4 knockdown combined with cisplatin. Wild-type p53 is known for its ability to induce apoptosis, the most important anti-tumor barrier [35]. However, p53 is the most frequently mutated gene in human cancers (more than 50%), and mutant p53 loses its ability to inhibit cell growth [36], and can inhibit cell apoptosis and induce carcinogenesis after exposure to DNA-damaging agents [37]. Our results show that mutant p53 expression is downregulated in MDA-MB-231 cells (harboring mutant p53). Moreover, the balance between the expression levels of pro-survival and pro-apoptotic Bcl-2 family members (e.g. Bcl-2 and Bax) is critical for cell survival or death [38, 39]. Our results show that combining CXCR4 knockdown and cisplatin treatment leads to upregulation of Bax, a pro-apoptotic protein and concomitant downregulation of Bcl-2, an anti-apoptotic protein, thereby decreasing the Bcl-2/Bax ratio compared with that in cells exposed to cisplatin alone. Most drug compounds reportedly induce apoptosis through intrinsic death signaling pathways. Caspase-3 is regarded as a member of the apoptosis executioner caspases and cleaves many key proteins in apoptosis [40]. We found that CXCR4 knockdown can increase the activation of caspase-3 and induce apoptosis. This indicates that CXCR4 knockdown may act through the Bax/Bcl-2/caspase-3 signaling pathway to induce apoptosis in MDA-MB-231 cells.

Studies have shown an association between cell cycle regulation and cancer, and inhibition of the cell cycle has become an appreciated target for cancer management [41]. In our study, CXCR4 knockdown augmented cisplatin-mediated G2/M phase arrest in MDA-MB-231 cells. The tumor suppressor p53 acts as a cell cycle checkpoint regulator, contributing to cell cycle arrest in the G1 and G2 phases via multiple pathways [42–44]. Activated p53 interacts with response elements present on the promoter region of p21 to increase expression of p21, which subsequently interacts with CDKs to affect cell cycle arrest [45, 46]. Since MDA-MB-231 cells express mutant p53 [47, 48], the downregulation of p53 that we observed by RNA interference-mediated knockdown of CXCR4 may increase cisplatin anti-tumor activity by inducing cell arrest in the G2/M phase.

*In vivo* experiments revealed that tumors in the cisplatin treatment group derived from MDA-MB-231-shCXCR4 cells show slower growth and smaller tumor volume. Data from the TUNEL staining assay show that the apoptosis index (AI) is dramatically increased in the CXCR4 shRNA combined with cisplatin group compared with the other three groups. Furthermore, we find the expression of mutant p53 and Bcl-2 decreases, and Bax proteins increase simultaneously.

In conclusion, our novel findings show that downregulation of CXCR4 can enhance cisplatin-induced growth inhibition and apoptosis in TNBC, suggesting that CXCR4 may be an emerging new strategy for TNBC therapy. These effects could be due to CXCR4 knockdown-induced G2/M arrest and apoptosis through upregulation of Bax and caspase-3 and downregulation of Bcl-2. Further studies should evaluate whether CXCR4-targeted therapy in combination with cytotoxic agents enhances the anti-tumor effects of chemotherapy in human clinical trials.

## MATERIALS AND METHODS

### Cell lines and cell culture

Triple-negative breast cancer cell lines MDA-MB-231 and MDA-MB-468 were purchased from the AmericanType Culture Collection (ATCC). All cells were grown in DMEM (Hyclone, Thermo Scientific, USA), supplemented with 13% fetal bovine serum, penicillin G (100 units/ml), and streptomycin (100 μg/ml)—termed complete medium—and maintained in monolayer culture at 37°C in humidified air with 5% CO_2_. We used a CXCR4 knockdown cell line, denoted MDA-MB-231-shCXCR4, previously described [13]. The cells were cultured at 37°C under a humidified 5% CO_2_ atmosphere.

### Transient transfections

Subconfluent proliferating MDA-MB-468 cells (a TNBC cell line, CXCR4 negative) were transfected with increasing amounts of pGCMV/IRES/EGFP CXCR4 expressing plasmid and control vector using Lipofectamine 2000 (Invitrogen) following the manufacturer's instructions. Twenty four hours after transfection, the cells line expressing CXCR4 (MDA-MB-468-CXCR4) or empty vector (MDA-MB-468-NC) was harvested for *in vitro* assay.

### Cell proliferation assay

The effect of cisplatin (Hansoh Pharmaceutical, Jiangsu, China) on the growth ability of TNBC cells was determined via MTT assay. Cells (5×10^3^ /well) were seeded in 96-well plates and incubated for 24 h in complete medium before the addition of increasing concentrations (0, 2.5, 5, 10, 20 and 40 μM) of cisplatin for 48 h. Then 20 μl MTT (5 mg/ml) was added to every well and incubated for 4 h. After removal of the medium, the dye crystals were dissolved in dimethyl sulfoxide (DMSO) and absorbance was measured at 492 nm with a Multiskan MK3 reader (Thermo Scientific). Inhibition was calculated as follows: IR (%) = [(absorbance of the control group – absorbance of the test group)/absorbance of the control group] × 100%. Three independent experiments were done in triplicate wells.

### Colony formation assay

Cells were seeded at a density of 1×10^5^ in a 24-well plate and allowed to adhere overnight. The cells were then treated with 0, 0.1 and 1 μM cisplatin. Forty-eight hours after cisplatin addition, cells were trypsinized, counted, and reseeded at a low density (200 cells/well in a 6-well culture dish) in triplicate. Medium was replaced every 3 days, and the cells were allowed to grow for 14 days. The number of colonies containing more than 50 cells in each dish was counted under a microscope. The cloning efficiency (CE) was calculated as follows: CE (%) = (number of colonies formed) / (number of cells added) × 100%.

### Cell cycle analysis

Cells were inoculated in 60 mm plates and incubated for 24 h to resume exponential growth. Cisplatin (0, 1 μM) was added and cells were incubated for an additional 48 h. Then the cells were harvested and washed twice with cold PBS and fixed in 70% ethanol (4°C). On the day of analysis, cells were collected by centrifugation. The pellets were resuspended in 0.1 mg/ml of propidium iodide (PI) containing 0.1% Triton X-100 and RNase A (1 mg/ml, both from Sigma, St. Louis, MO, USA). The cell suspension was incubated in the dark for 30 minutes at room temperature. Cell cycle and apoptosis were quantified by measuring the DNA content of cells by flow cytometry (BD, FACSCalibur, USA). The proportion of sub-G1 phase (apoptotic) cells (%) and the proportions cells in other phases (%) reflected cell cycle. The experiment was repeated three times. The percentage of cells in different phases of the cell cycle was determined using a ModFit 3.1 computer program.

### Western blot assay

Both cell lines, at a density of 1×10^5^ /ml, were exposed to 10 μM cisplatin for 48 h. Cells not treated with cisplatin were used as controls. Cells were collected with a cell scraper and placed in lysis buffer for 30 min at 4°C, then centrifuged at 12,000 g at 4°C for 15 min. The supernatant was then collected. Total protein was quantified using a BCA kit (Beyotime, China), and 30 μg of protein was separated by 12.5% SDS-PAGE and transferred onto a polyvinylidene difluoride membrane (Millipore, Billerica, MA). Membranes were blocked using 5% non-fat milk in TBST for 2 h at room temperature. After briefly washing with 0.1% Tween 20 (Sigma, St. Louis, MO, USA) in TBS, the membrane was incubated with anti-CXCR4 (ab2074, Abcam Corp. Cambridge, United Kingdom), anti-p53 (BM0101, Boster, Wuhan, China), anti-caspase-3 (#9665, Cell Signaling Technology, Beverly, MA), anti-Bax (#5023, Cell Signaling Technology, Beverly, MA), anti-Bcl-2 (#4223, Cell Signaling Technology, Beverly, MA) and anti-β-actin (TA-09, Zhongshan Golden Bridge Biotechnology, Beijing, China) antibodies at 4°C overnight. Blots were then incubated in secondary antibody conjugated with HRP (Santa Cruz Biotechnology, CA) for 2 h at room temperature. The protein bands were visualized with ECL plus Western blotting detection reagents (Thermo Scientific, USA).

### Animals

All animals were cared for in accordance with the Animal Welfare Act guidelines under an animal protocol approved by Shantou University Medical College Animal Care and Use Committee. Six-week-old female BALB/c nude mice (Vital River Laboratories, Beijing, China) were used to establish the experimental orthotopic nude mouse model.

### Orthotopic nude mice breast tumor model

For the experimental orthotopic model, sub-confluent MDA-MB-231-NC or MDA-MB-231-shCXCR4 cells were harvested by trypsinization and resuspended in DMEM. Only single-cell suspensions with 95% viability were used. Cells (5 × 10^6^) were inoculated into bilateral breast fat pads (orthotopic tumor model), with the left side being inoculated with MDA-MB-231-NC cells and the right side with MDA-MB-231-shCXCR4 cells. When tumors reached a size of approximately 100 mm^3^, animals were then randomly allocated for cisplatin treatment or 0.9% saline. Then mice were intraperitoneally injected with 2 mg/kg cisplatin for one week, while the control group received an equal volume of 0.9% saline and the treatment was administered every third day. The tumor sizes were measured every three days using a digital caliper. The tumor volume were determined with the formula: tumor volume [mm]^3^ = (length [mm]) (width [mm])^2^ ×0.52. The average tumor volume of each group was calculated and tumor growth curves were drawn accordingly. The rates of anti-tumor activity were then calculated. After 3 weeks, all surviving mice were euthanized by an overdose of CO_2_ exposure and were evaluated macroscopically for the presence of orthotopic tumors. After treatment, the tumor xenografts were excised and weighed. Tumor tissues were fixed in 10% buffered formalin, embedded in paraffin, cut into 4-μm-thick sections, and then used for the TUNEL and immunohistochemical assays.

### Immunohistochemistry

Orthotopic tumor paraffin sections were dewaxed and rehydrated, and antigen retrieval was performed by microwaving in 10 mM sodium citrate buffer, pH 6.0, for 15 minutes. Sections were then incubated with 3% hydrogen peroxide for 20 min at room temperature to block endogenous peroxidase, then blocked in 10% normal goat serum for 1 h. Immunostaining was performed by incubating with anti-PCNA (bs-2006R, Bioss, Beijing, China) antibody, anti-p53 antibody, anti-caspase-3 (ZM-0320, Zhongshan Golden Bridge Biotechnology, Beijing, China) antibody, anti-Bax antibody or anti-Bcl-2 antibody at 4°C overnight. Slides were then washed in PBS and incubated with biotinylated secondary antibody (GTVision I, Anti-Mouse/Rabbit Detection System, Gene Tech Company) for 30 min at 37°C. Staining was visualized with 3, 3-diamino-benzidine (DAB) and counterstained with hematoxylin.

### Apoptotic index (AI)

Detection of apoptotic cells was performed on the basis of the TUNEL method with an ApopTag Peroxidase *in situ* cell apoptosis detection kit (Boster, Wuhan, China) according to the manufacturer's directions. Tumor sections were examined by microscopy. At least 100 tumor cells per field were counted in five randomly selected fields at a 40× magnification. Cells in which the nucleus or cytoplasm was dyed yellow and brown were identified to be undergoing apoptosis. The percentage of apoptotic cells was calculated as the number of apoptotic cells divided by the total number of tumor cells. Two individuals who were blinded to the treatment group counted cells independently. The apoptotic index was determined from the average of counts from five randomly selected fields.

### Statistical analysis

Each assay was performed in triplicate and repeated a minimum of three times. Statistical analysis was performed using SPSS 17.0 for Windows. Data are reported as means ± *SD*. Statistical differences were analyzed by Student's *t*-test for paired data between control and treated groups, or a one-way analysis of variance (ANOVA) for data from multiple groups, with the level of significance set at *p* < 0.05.
