# Correction: Silencing of CXCR4 sensitizes triple-negative breast cancer cells to cisplatin

**DOI:** 10.18632/oncotarget.28109

**Published:** 2022-04-01

**Authors:** Sixian Liang, Xun Peng, Xiaoli Li, Ping Yang, Linhao Xie, Yaochen Li, Caiwen Du, Guojun Zhang

**Affiliations:** ^1^Department of Breast Medical Oncology, Cancer Hospital of Shantou University Medical College, Shantou 515031, PR China; ^2^Department of Radiotherapy, Cancer Hospital of Shantou University Medical College, Shantou 515031, PR China; ^3^The Breast Center, Cancer Hospital of Shantou University Medical College, Shantou 515031, PR China; ^*^These authors have contributed equally to this work

**This article has been corrected:** In Figure 4C, panel ‘o’ contains a partial accidental overlap of panel ‘e’. The corrected Figure 4, produced using the original data, is shown below. The authors declare that these corrections do not change the results or conclusions of this paper.

Original article: Oncotarget. 2015; 6:1020–1030. 1020-1030. https://doi.org/10.18632/oncotarget.2741

**Figure 4:**
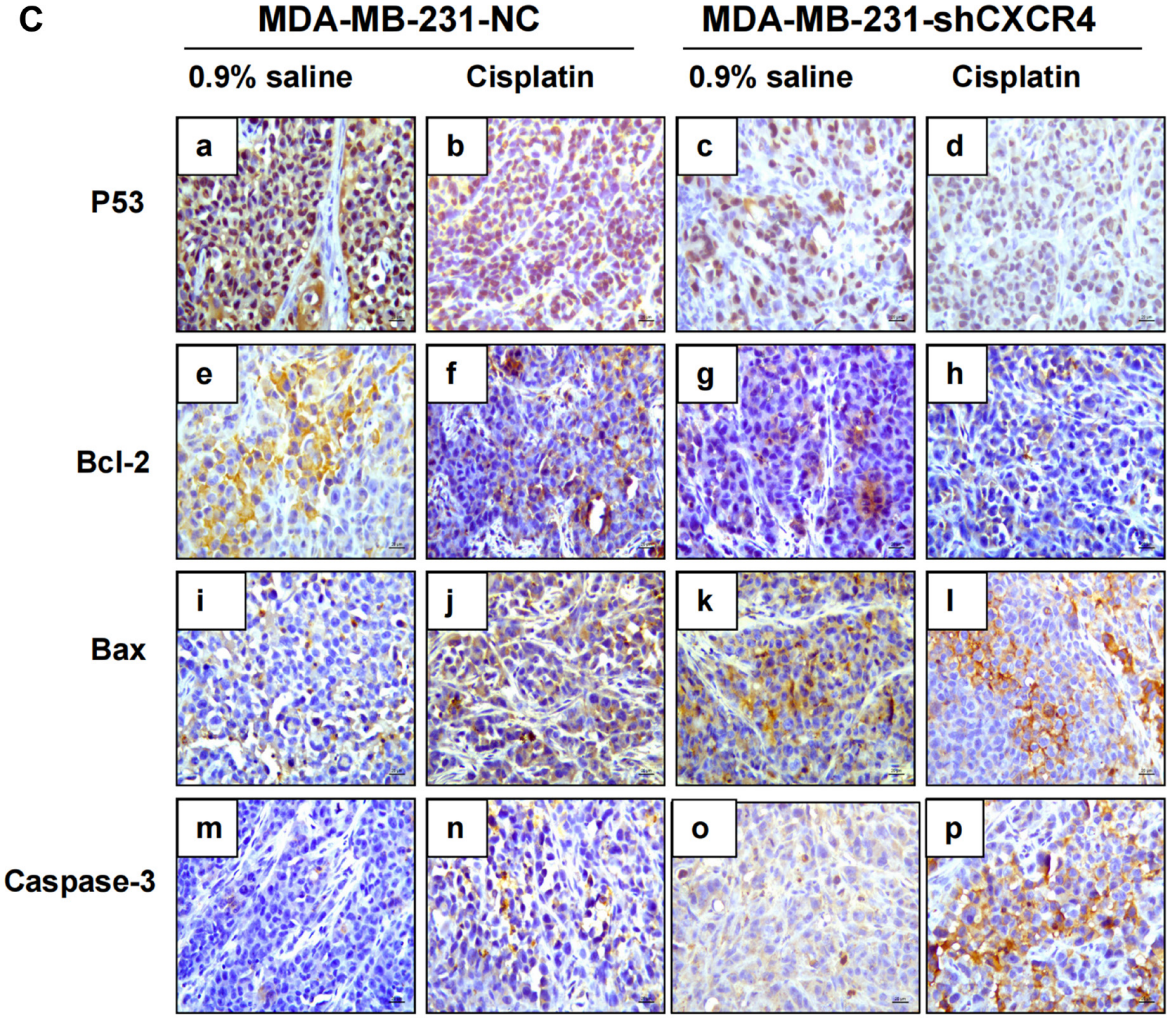
(**C**) Immunohistochemical staining of xenograft tumors for p53, Bax, Bcl-2 and caspase-3 (magnification = 40×).

